# Chicago Biological Society

**Published:** 1882-11

**Authors:** 


### ARTICLE X.

THE CHICAGO BIOLOGICAL SOCIETY. Stated Meeting, October 4, 1882.

Dr. Lester Curtis, president *pro tem.*

### MINERAL ORGANISMS AND THE ORIGIN OF LIFE.

That was the title of a paper by Dr. H. D. Valin, who repeated experiments in organic forms before the society. In January of the present year, the French chemists, D. Monnier and C. Vogt presented, through M. Robin, to the French Academy of Sciences, the results of some experiments, showing that the forms-peculiar to plants and animals also appear under certain circumstances in purely inorganic things. (Comptes Rendus, Jan. 2, 1882). This is their language: "Objects endowed with well-defined shapes, exhibiting all the characteristics of the forms met in organic bodies, such as simple cells, cells with porous tubes attached, tubes with walls or with partitions, filled with heterogenous and granular contents, etc., can be artificially produced in an appropriate liquid by the reactions of the salts, forming, by double decomposition, either two, or one insoluble salt. One of these salts must be dissolved in the liquid, while the other must be solid in form. * * * * The forms met in organic bodies (cells and tubes) being produced just as well in a liquid with an organic or semi-organic (sucrate of calcium) origin, as in a liquid of a purely inorganic origin (silicate of sodium), there cannot be henceforth any characteristic forms by which to distinguish inorganic bodies on the one hand, from organic bodies on the other. * * * * It is likely that the inorganic substances met in organic protoplasm are for something in determining the forms which living organisms assume."

Dr. Valin had repeated these experiments a number of times in the last six months, and made the following observations: In a flask full of soluble glass, were placed fragments of sulphate of iron, ten grains in weight, which immediately began to assume a colloid condition on the outside, and shot tubular prolongations, colloidal and cellular, which grew at the rate of half an inch in twenty-four hours. Some attained to two-inches in length, and were about $\frac{1}{12}$ of an inch in diameter. All these prolongations shot a number of slender filaments from various points of their surface, and these attained a length of a few inches in a few hours. After a few days or weeks all these organisms assume a crystalline condition and become empty inside. Some of them rise to the surface of the liquid. They are insoluble in water, they remain intact when exposed to air, and when introduced in a newly prepared flask at the same time with fresh fragments, they hasten the metamorphosis of these. The addition of water to the soluble glass renders the experiments more easy and saves time.

Watched under the microscope, the fragment of sulphate of iron is seen to swell all around. An unctuous, colloid mass is formed, which consists of five granules perfectly similar to animal tissues. This mass stretches into prolongations, and fluid contents are seen to flow inside these. When the surface of some prolongations was opened into, a semi-solid substance grew out of the opening into new prolongations. One of these mineral organisms, when placed on a fresh fragment shot some new prolongations. as if real grafting had taken place.

Organisms of sulphate of copper, sulphate of zinc, alum, phosphate of iron, etc., were similarly obtained, each possessing a form peculiar to itself, and distinct from the others. Analogous forms grew in saccharated lime-water. Cellular bodies of the same minerals formed in solutions of alkaline carbonates.

These experiments relate to the almost unknown department of chemistry which treats of the colloids, and as crystalline solutions grow into symmetrical crystals, so a colloid substance in process of formation assumes a typical form, and must be the start of all forms in animals and plants. These so-called mineral organisms, viewed with the naked eye, under the microscope, or chemically tested, come as near to the lower animals and plants as these are from one another, and form a new field of investigation for the biologist. We can no longer say that only living things grow, unless we reckon these as living.

The discovery of these organisms has attracted the attention of the scientific world, the more readily since Herbert Spencer, in his biology, made the following suggestion concerning the origin of life: "The colloid is, in fact, a dynamical state of matter, the crystalloidal being the statical condition. The colloid possesses *energia.* It may be looked upon as the primary source of the force appearing in the phenomena of vitality." (Biology 56.)

Among the conclusions of Dr. Valin's paper were these: "That the vitality or growth of these mineral organisms consists in the passage of a crystal into a colloid, and is thus correlated, but not identical, with the kinetec process known as crystallization. That the molecule of these bodies consists of many elements, and that acid and alkaline polarities are always concerned in their growth, for only acid minerals in alkaline solutions gave rise to them. That we have a right to suppose that living protoplasm is nothing but a highly complex mineral organism in favorable media (water and air)."

This would tend to confirm the growing belief among biologists that life is nothing but the energy manifested by the forty and odd (Reinke) proximate principles which constitute protoplasm, when they pass from the crystalline or soluble into the colloid state in the proper media.

In the brief discussion which followed, Dr. Clevenger asked the writer whether he believed that the growth of these minerals might not be dependent on the action of some micro-organism.

Dr. Valin answered that some micrococci had been seen in the solutions used, and that a large fungus at one time covered the surface of the water in one flask with its mycelium, visible to the naked eye. But as the minerals referred to grow instantaneously in any kind of water, and as this water remains transparent, it excludes the possibility of any bacterial action. The question was the more pertinent, however, as Alphonse Wurtz, the great chemist, made a communication to the Académie des Sciences not long ago, in which he described a vibrion which is always found in certain germinating seeds, as that of Indian corn. It seems that this micro organism is indispensable to the process of germination and that its role consists in eating into the outer coat of the seed, which thus becomes permeable to water.

---

In a village in Austria there was the marriage of a rich farmer's daughter with the only son of another rich farmer. Three days and three nights the eating and drinking continued, and then all the participants of the feast took to the wedding cake. But unfortunately, this cake was poisoned, and one person died, and many others became very sick. So the court ordered the rest of the cake to be examined by the county physician. This gentleman, putting the cake, carefully wrapped up, into his overcoat, went home—a little later than usual. The next morning the cake was gone, but the physician had great difficulty in saving his wife from being poisoned.

